# Supplementary material for: HPK1 citron homology domain regulates phosphorylation of SLP76 and modulates kinase domain interaction dynamics
Source: Nat Commun. 2024 May 2;15:3725. doi: 10.1038/s41467-024-48014-9 (PMC11066036; doi:10.1038/s41467-024-48014-9)
Supplement: Supplementary file 1 — Supplementary Information [file 41467_2024_48014_MOESM1_ESM.pdf]

Supplementary Information

HPK1 Citron Homology Domain Regulates Phosphorylation of SLP76 and Modulates Kinase Domain Interaction Dynamics

Authors: Avantika Chitre, Ping Wu, Benjamin T. Walters, Xiangdan Wang, Alexandre Bouyssou, Xiangnan Du, Isabelle Lehoux, Rina Fong, Alisa Arata, Joyce Chan, Die Wang, Yvonne Franke, Jane L. Grogan, Ira Mellman, Laetitia Comps-Agrar, Weiru Wang

Supplementary Figures

Supplementary Figure 1

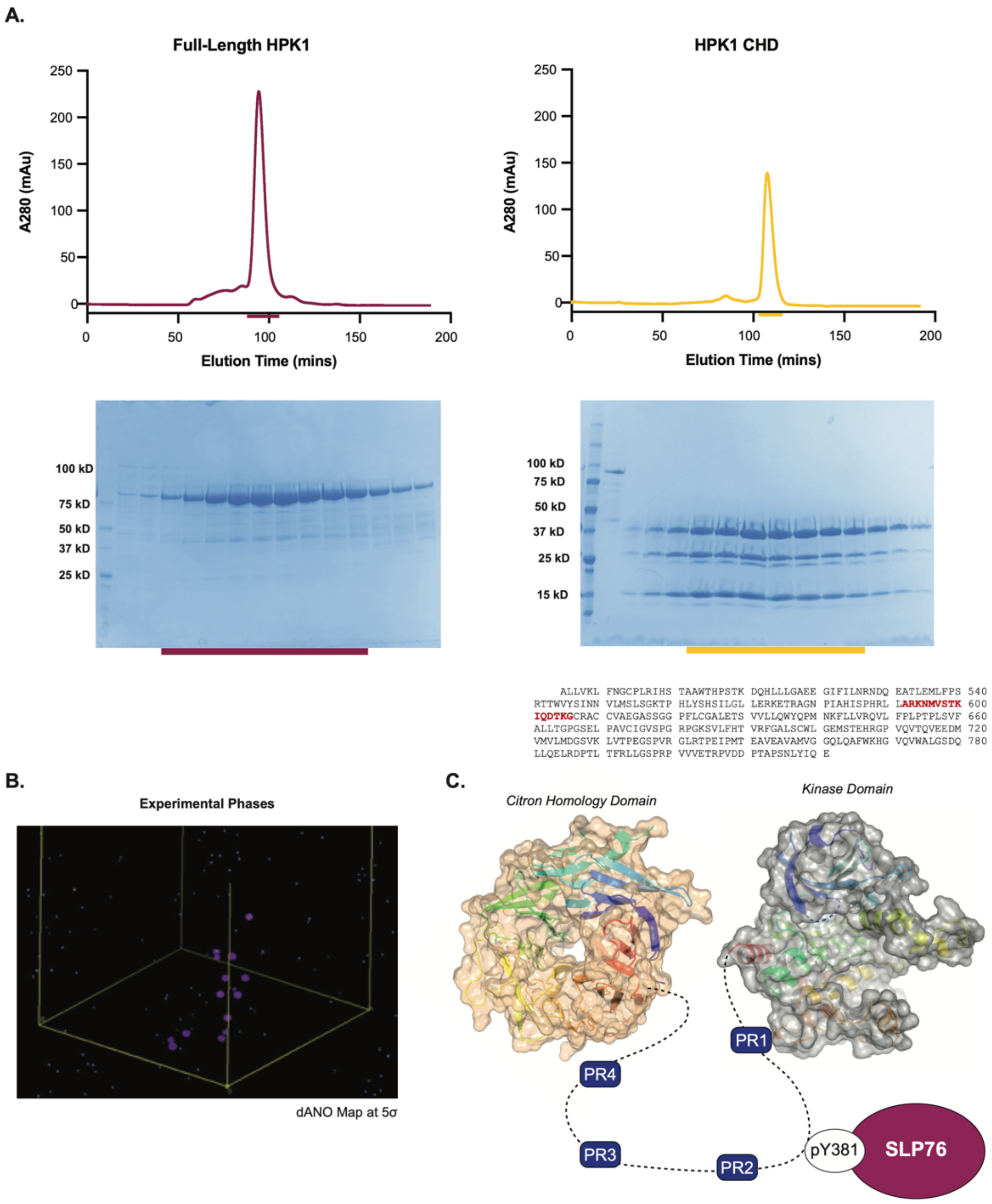

(A) The size exclusion chromatography (SEC) profile and SDS-PAGE of purified HPK1 full (top left) and CHD (top right). The SEC profile indicates homogeneously structured protein structure. SDS-PAGE indicates CHD is partially cleaved as a specific site, while full-length HPK1 is not cleaved. Commassie

Blue gels (FL HPK1 bottom left), HPK1 CHD bottom right) demonstrating sizes of protein components of each peak. (B) The CHD structure was solved by single-wavelength anomalous diffraction (SAD) phasing method using Selenomethionine (SeMet) as the heavy atoms. The purple mesh shows difference-anomalous (dANO) map contoured at 5.0 σ, depicting the Se atom positions used for calculating experimental phases. (C) A model of domain architecture of full-length HPK1 and interacting proteins. The citron homology domain and kinase domain are crystal structures. The proline-rich (PR) region structures are unknown, therefore depicted in a dotted line. Four PR regions (shown in boxes) bind to adaptor proteins. The position of Tyr381 is flagged in a circle. Phosphorylation of Tyr381 is critical for SLP76 engagement. (D) A portion of the 2fo-fc electron density countered at 1.0 σ.

Supplementary Figure 2

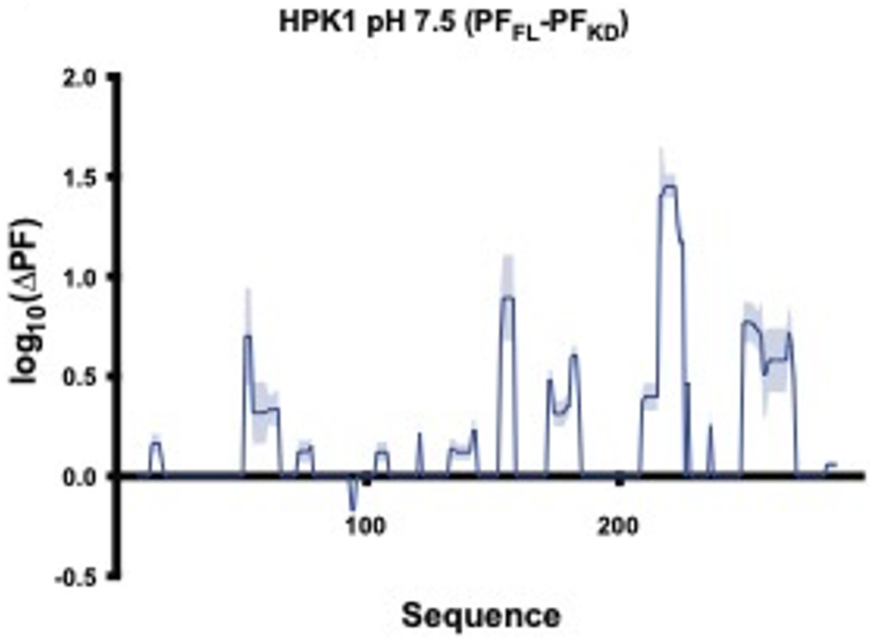

Extended Data for Hydrogen-Deuterium Exchange Mass Spectrometry. Protection factors expressing the difference in exchange observed for full length HPK as compared to the KD alone (n=3). A positive value represents reduced exchange in the full-length protein. Shading on the line represents the uncertainty in that value as estimated by an empirical method, introduced previously.[41]

Supplementary Figure 3

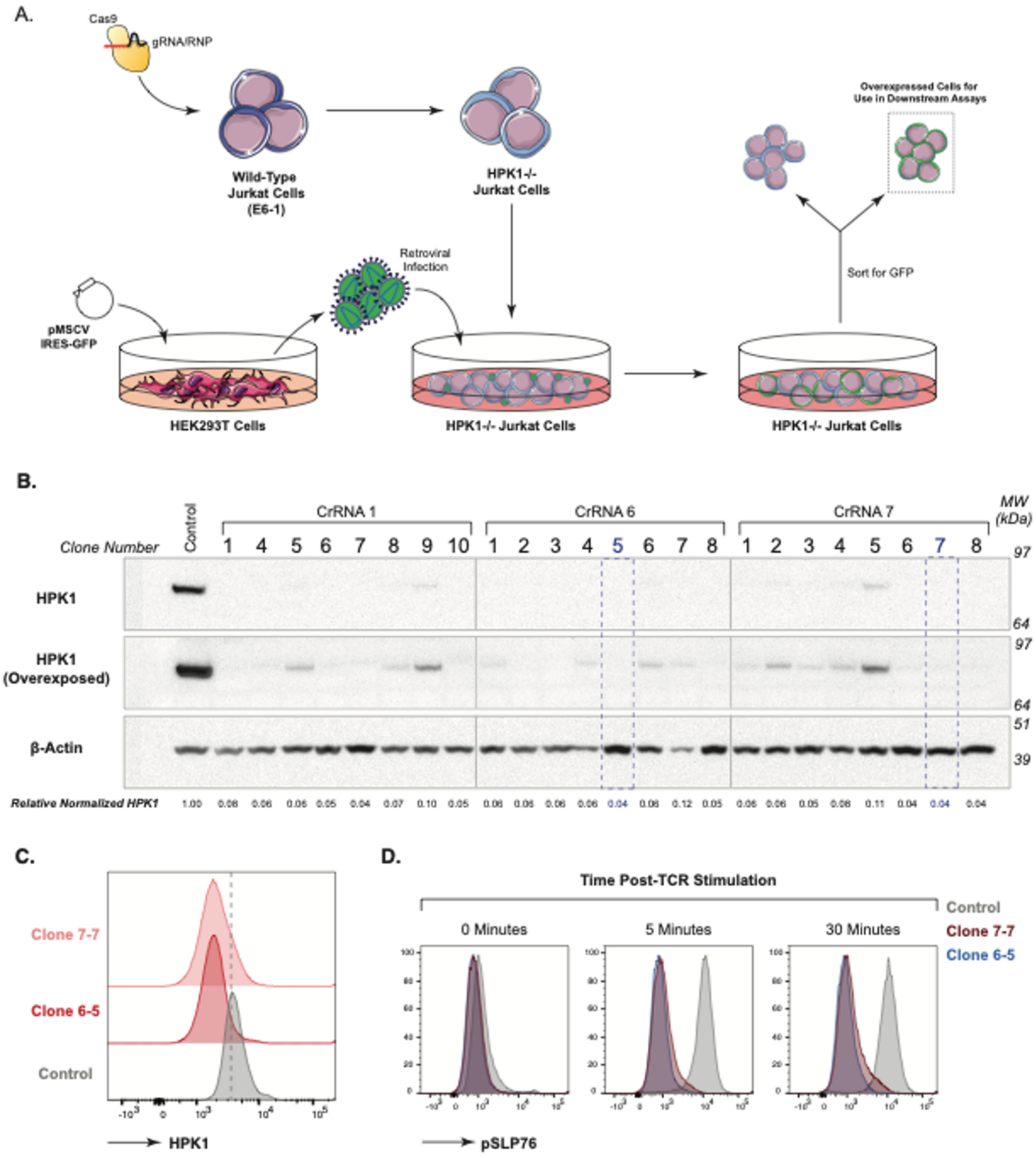

Generation of HPK1 Knockout Cells and Method for Protein Overexpression. (A) Schematic demonstrating process of generation of HPK1-/- Jurkat cells and expression of HPK1 forms encoded by pMSCV constructs using a retroviral expression method. (B) Western blot for HPK1 protein in clonal populations of cells knocked out for HPK1 using CRISPR. populations with the lowest protein expression were selected for downstream assays. (C) Assessment of protein expression by flow cytometry for clonal populations selected from (B). (D) Measurement of functional HPK1 remaining in CRISPR-generated HPK1-/- clonal populations via a flow cytometry-based phospho-SLP76 assay over a 30-minute timecourse after anti-CD3/CD28 stimulation. Data from (B-D) is from a single experiment.

Supplementary Figure 4

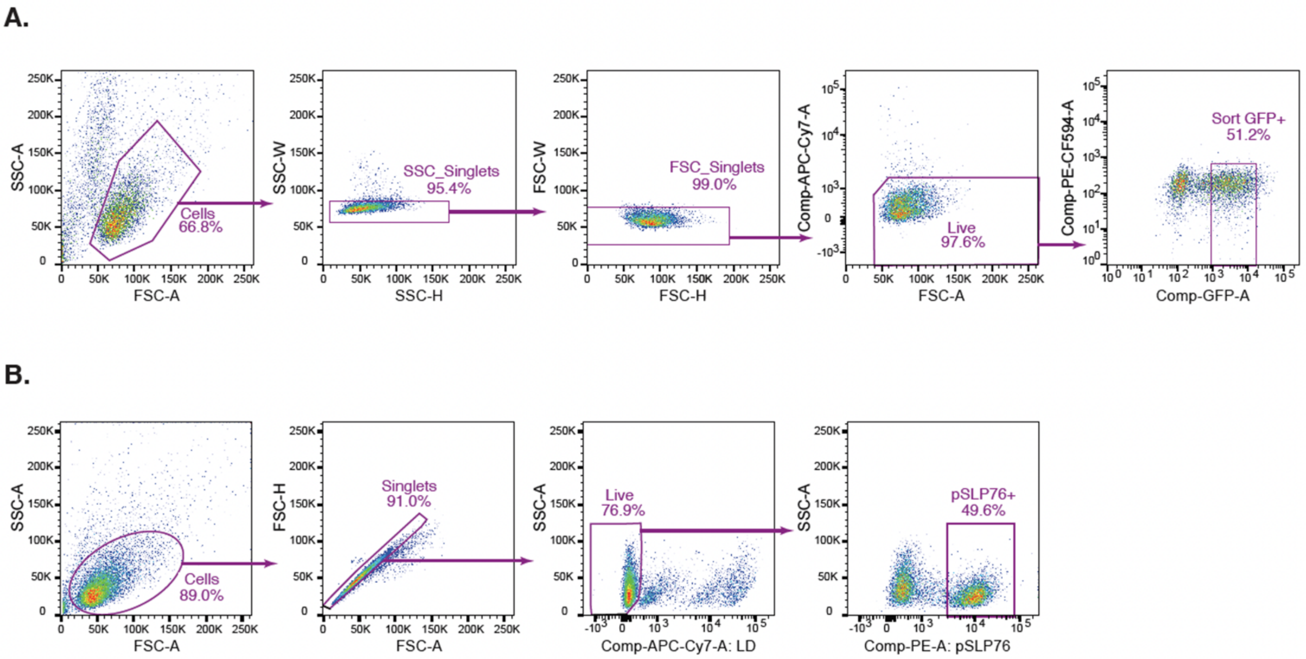

Gating Strategy for Purification of Retrovirus-Expressing HPK1-/- Jurkat Cells and Assessment of Phosphorylated SLP76 by Flow Cytometry. (A) Representative gating strategy for sort-based enrichment of construct expressing HPK1-/- Jurkat cells, sorting for GFP+ cell populations. (B) Representative gating strategy for flow-based assay measuring amount of phosphorylated SLP76 in construct-expressing HPK1-/- Jurkat cells.

Supplementary Figure 5

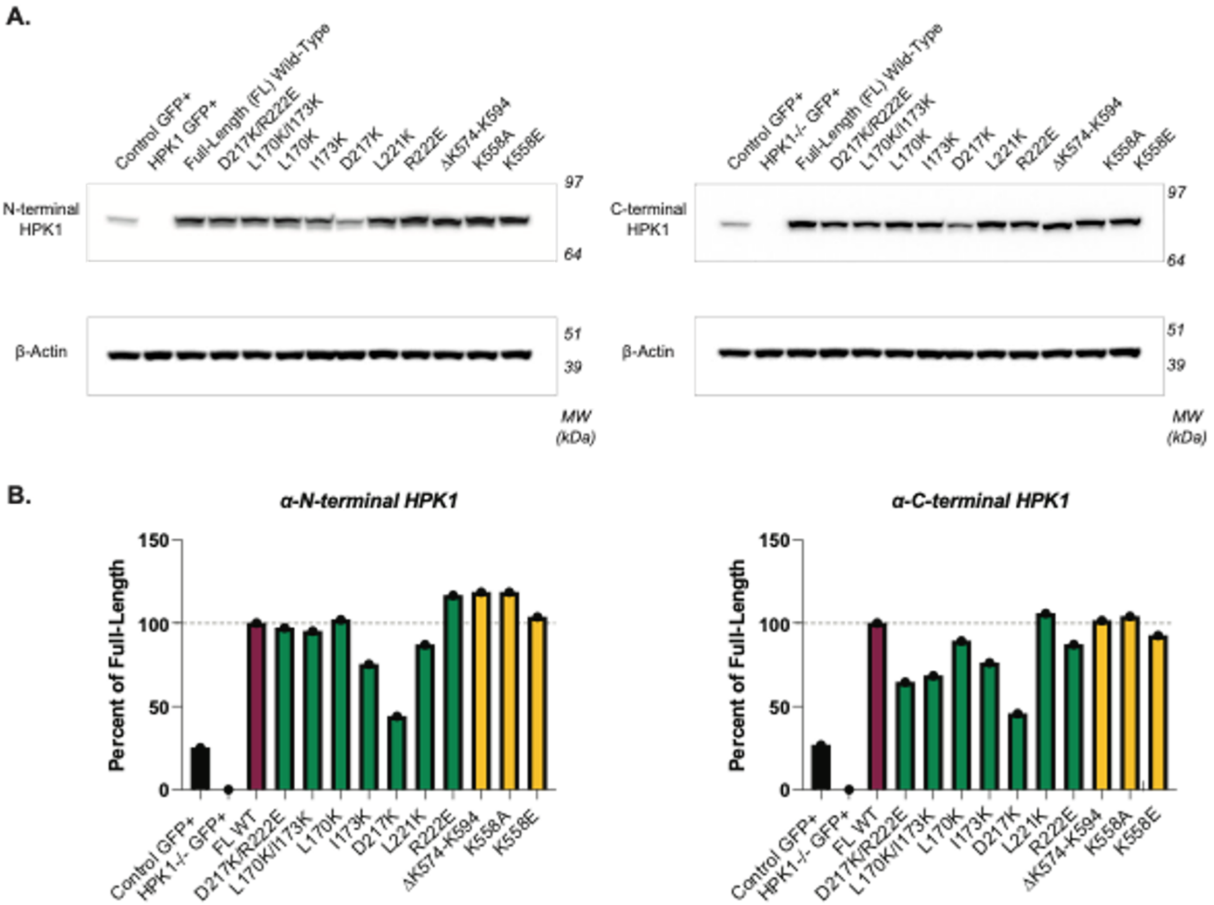

(A) HPK1 protein expression as measured by western blot probing for N-terminal (left) or terminal (right) HPK1 in HPK1-/- Jurkat cells expressing the indicated mutant form of HPK1. (B) Quantification of normalized HPK1 expression from western blots in (A). Data shown in (A-B) is a representative experiment of three independent experiments (n=3), with means ± S.D. displayed in (B).

Supplementary Figure 6

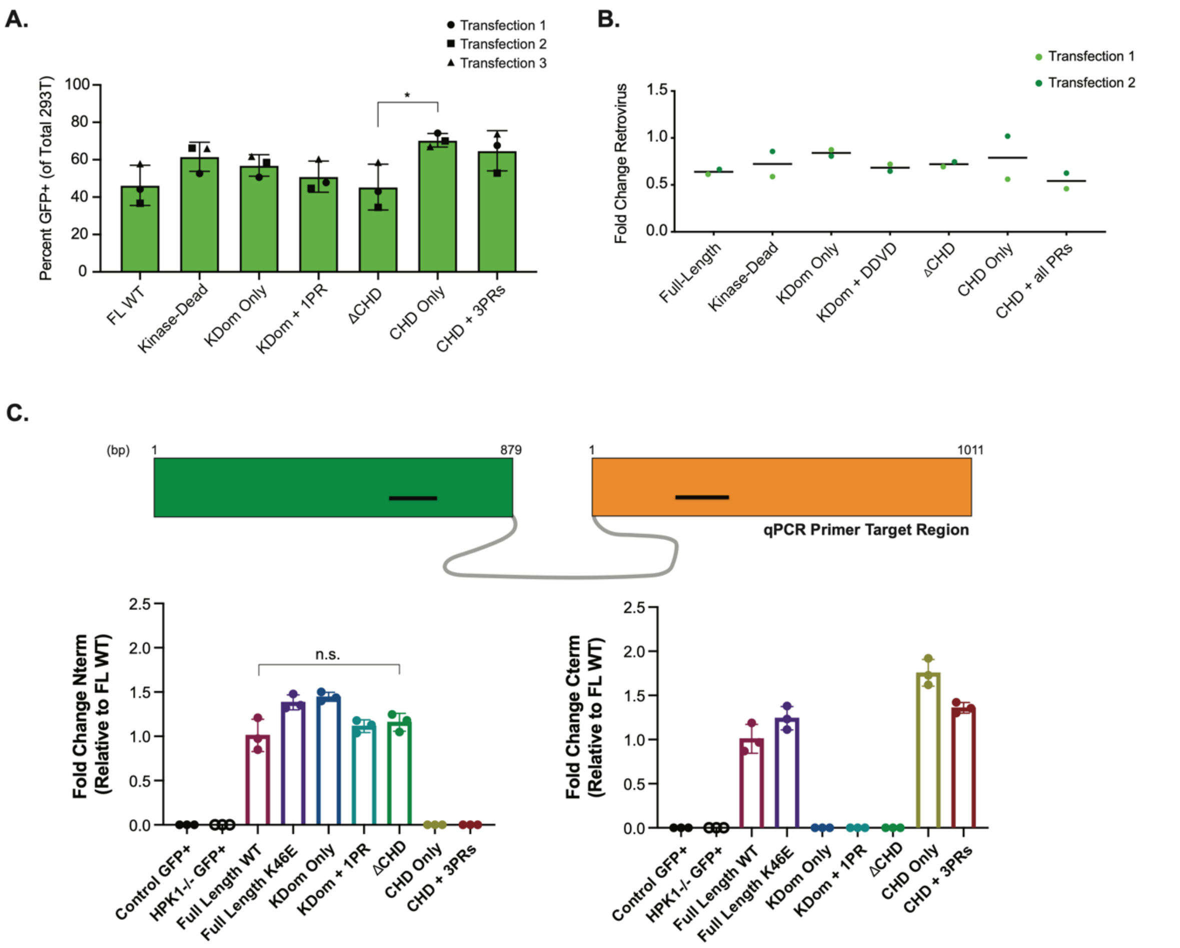

Reduced Protein Expression of ΔCHD is Not Due to Protocol Discrepancies or Transcriptional Regulation. (A) Transfection efficiency across all constructs, as measured by percent GFP+ cells of 293T cells 48 hours after transfection. Rounds of transfection are indicated by symbol shape. Data shown is a representative experiment of three independent transfections (n=3). Means ± S.D. are displayed. Statistical significance was determined using a one-way ANOVA. *p=0.0479 (B) Comparison of virus production efficiency, determined by quantifying the amount of retrovirus in supernatant from HEK 293T cells transfected with the indicated construct 48 hours post-transfection. Rounds of transfection are indicated by symbol color. Data shown is a representative experiment of two independent transfections (n=2) with means ± S.D. displayed. (C) Transcript levels of N-terminal (left) or C-terminal (right) HPK1 in Jurkat cells expressing the indicated form of HPK1 after retroviral infection. Sequence of HPK1 that is

targeted by the qPCR probe is indicated on the schematic of HPK1 above. Data shown is an experiment from a single transfection completed in technical triplicate (n=3) with means ± S.D. displayed. Lack of statistical significance between full-length versus ΔCHD was determined using a one-way ANOVA with Dunnett's multiple comparisons test. n.s.= not significant

Supplementary Figure 7

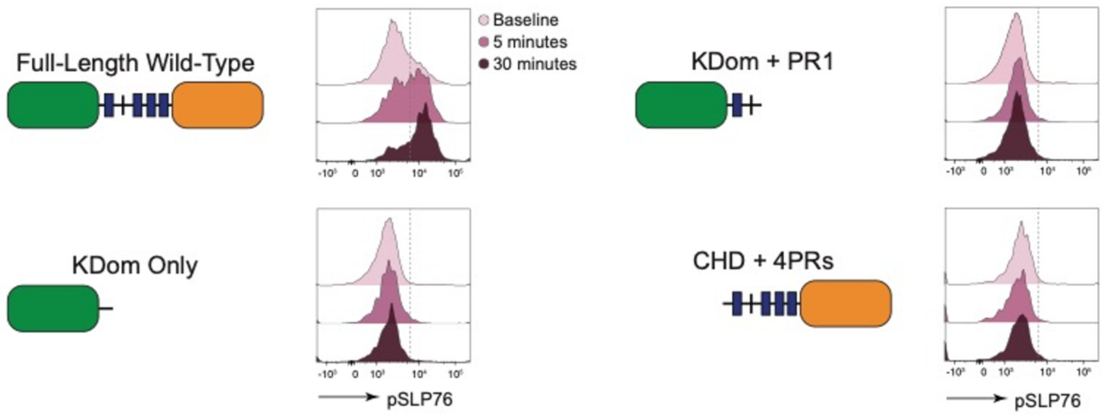

Phosphorylation of SLP76 over a 30-minute time course after TCR stimulation in Jurkat cells expressing the indicated version of HPK1. Data shown is one representative experiment of three independent experiments with technical triplicates (n=3).

Supplementary Figure 8

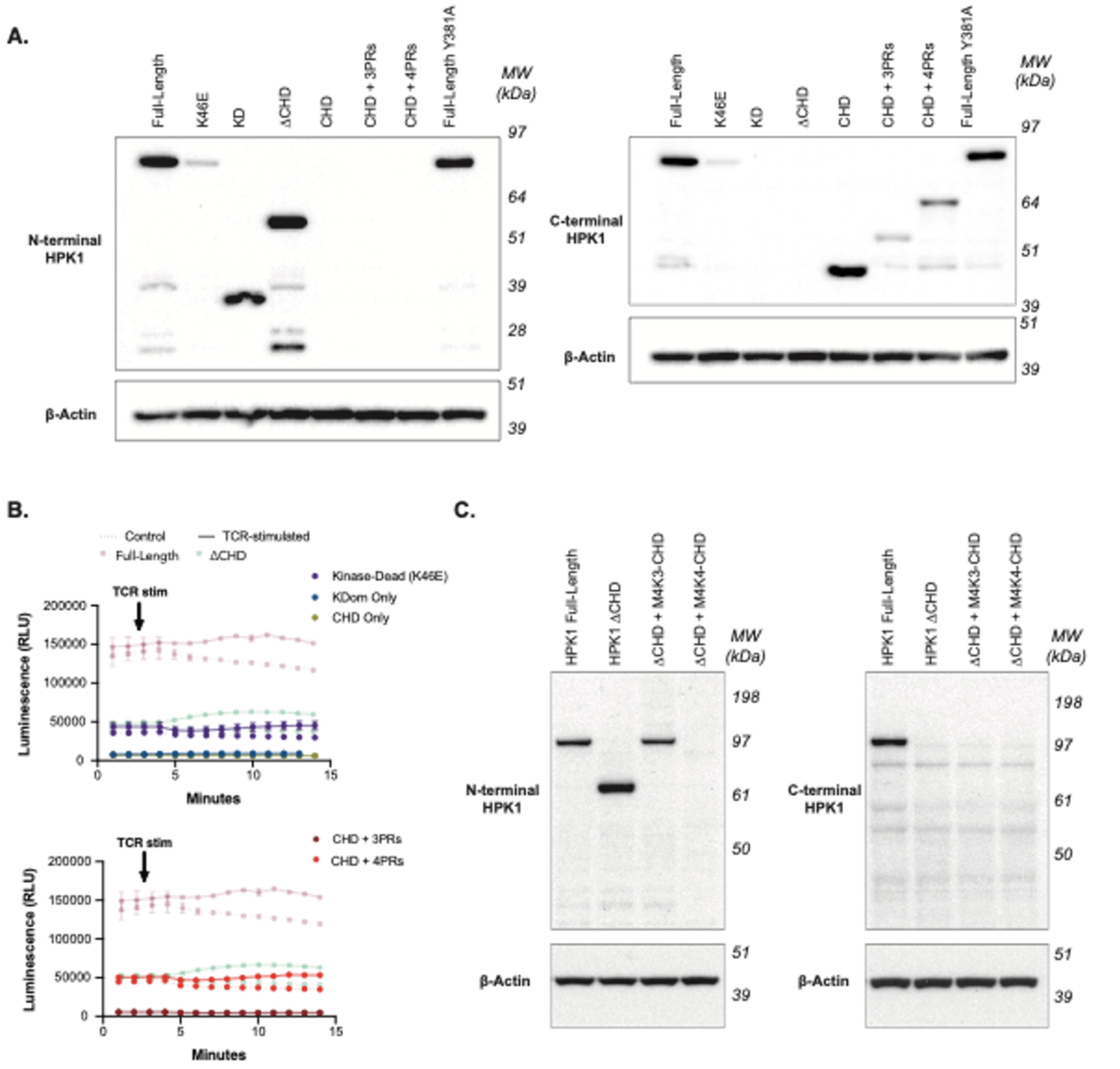

Recruitment of SLP76 to HPK1 in Live Cells. (A, C) Expression of the indicated forms of HPK1-LgBit as assessed by Western blot analysis 24 hours post-transfection. Antibodies against N-terminal HPK1 (left) or C-terminal HPK1 (right) were used for the immunoblot. Data are representative of three independent transfections and protein assessment experiments. (B) NanoBit technology was used to measure the interaction in real time between the indicated truncated or mutated forms of HPK1-LgBit and SLP76-SmBit expressed in transfected Jurkat HPK1-/- cells. Luminescent signals were recorded on cells stimulated with CD3/CD28 antibodies (solid lines) or isotype control (dotted lines). Arrow indicates time of

TCR stimulation. Representative data of three independent experiments, each performed in triplicates (n=3), are shown as means ± S.D.

Supplementary Figure 9

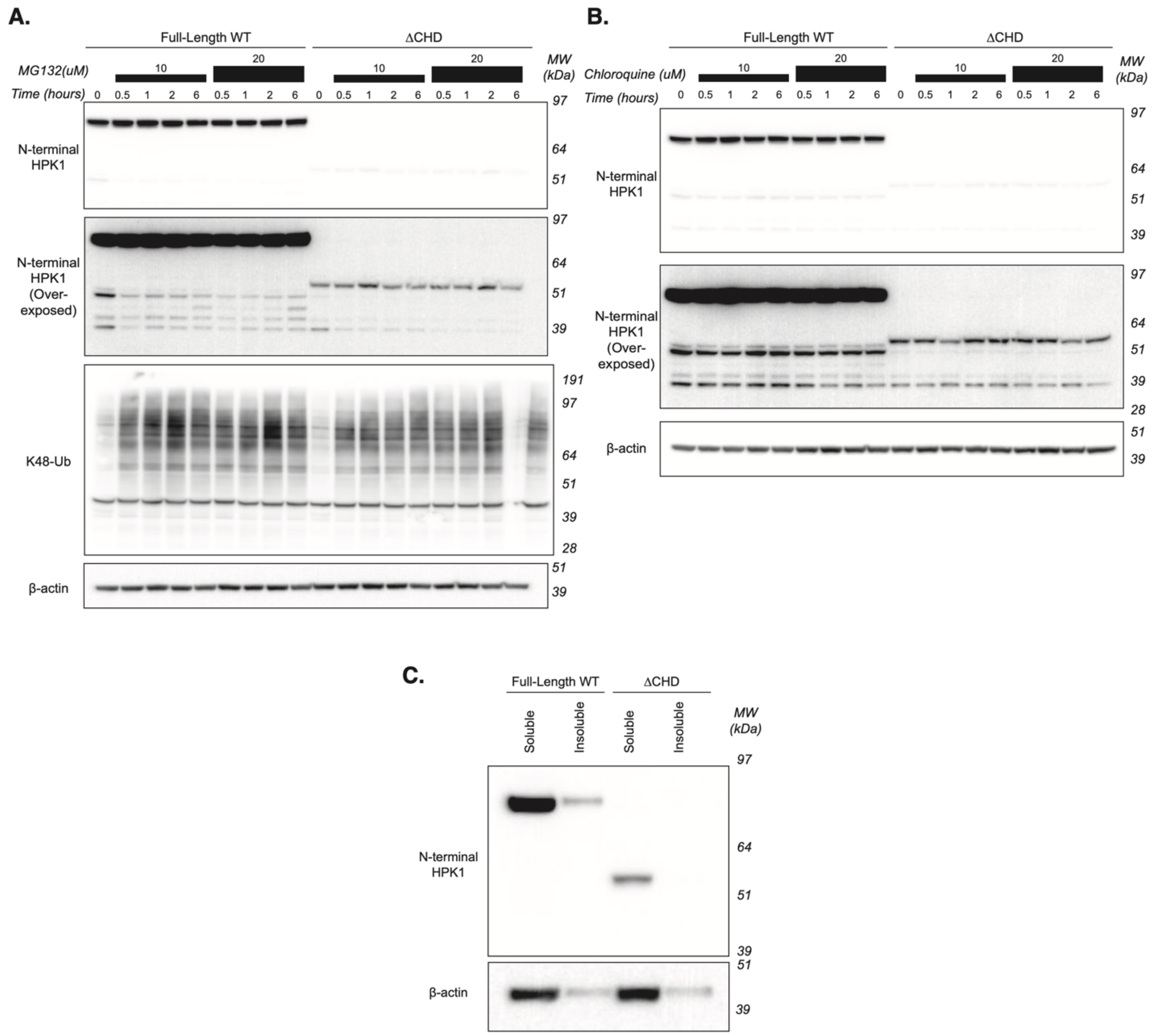

ΔCHD Does Not Appear to Be Degraded via the Proteasome, Lysosome, or Unfolded Protein Response. (A) Assessment of proteasome degradation in Jurkat cells expressing full-length (left) or ΔCHD (right) via treatment with MG132 (10 or 20 μM) over a 6 hour timecourse by Western Blot. Data shown is representative of two independent experiments. (B) Measurement of lysosomal degradation in full-length (left) or ΔCHD (right) by treating cell lines with lysosomal inhibitor chloroquine (10 or 20 μM) over a 6 hour timecourse by Western Blot. Data shown is representative of two independent experiments. (C)

Measurement of amount of protein aggregates as a surrogate of misfolded protein in soluble (NP-40) or insoluble (NP-40 + SDS) lysate fractions from cells expressing either full-length (left) or ΔCHD (right) HPK1. Data is from a single experiment.

Supplementary Figure 10

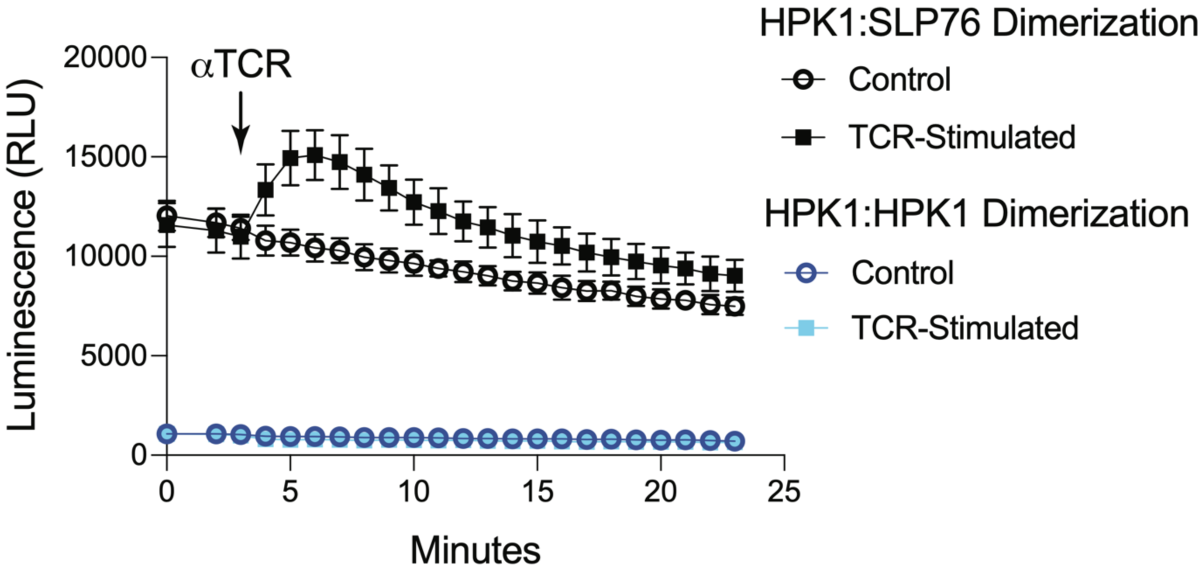

Assessment of Full-Length HPK1 dimerization. HPK1 -/- Jurkat cells were transfected with constructs expressing SmBit and LgBit HPK1 to assess HPK1 dimerization in real time using NanoBit technology. HPK1:SLP76 was used as a positive control for interaction. Luminescent signals were recorded on cells stimulated with CD3/CD28 antibodies (solid lines) or isotype control (dotted lines). Arrow indicates time of TCR stimulation. Data represents means ± S.D. of triplicates of one experiment (n=1).
