# Supplementary material for: HPK1 citron homology domain regulates phosphorylation of SLP76 and modulates kinase domain interaction dynamics
Source: Nat Commun. 2024 May 2;15:3725. doi: 10.1038/s41467-024-48014-9 (PMC11066036; doi:10.1038/s41467-024-48014-9)
Supplement: Supplementary file 3 — Description of Additional Supplementary Files [file 41467_2024_48014_MOESM3_ESM.docx]

**Description of Additional Supplementary Files**

**Supplementary Data 1:** Contains all timepoints for all peptides measured in the KD to Full Length experiments. Error bars represent the standard deviation of triplicate measurements.

**Supplementary Data 2:** Contains all timepoints for all peptides measured in the CHD to Full Length experiments. Error bars represent the range of duplicate measurements.

**Supplementary Data 3:** (“Data_table” Tab) General information about the experiments, including replicates and degeneracy, etc. (“Protection Factors (Kin --> FL)” Tab) Protection factors observed from kinase to full length, containing all information for peptides used in the final analysis: (a) Deuterium Overlap: this means the amount of y-axis uptake that overlapped between the experimental conditions, used for empirical PF analysis; (b) Significant log10(PF): the log10 empirical protection factor measured. (c) log10(PF)uncertainty: the uncertainty estimate obtained from errors at each uptake point in relation to the empirical PF measurement
