# Supplementary material for: HPK1 citron homology domain regulates phosphorylation of SLP76 and modulates kinase domain interaction dynamics
Source: Nat Commun. 2024 May 2;15:3725. doi: 10.1038/s41467-024-48014-9 (PMC11066036; doi:10.1038/s41467-024-48014-9)
Supplement: Supplementary file 4 — Supplementary Data 1 [file 41467_2024_48014_MOESM4_ESM.pdf]

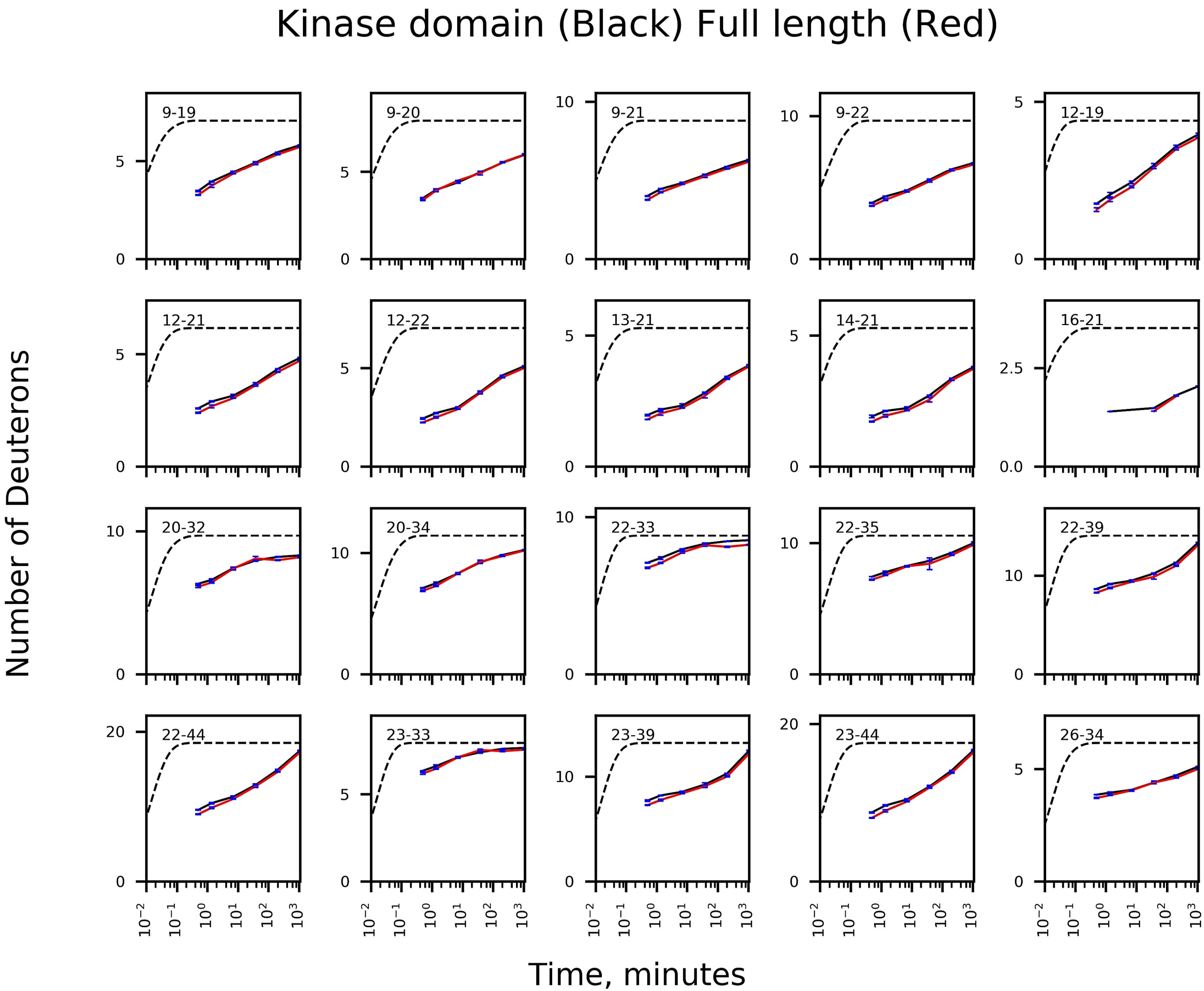
Kinase domain (Black) Full length (Red)
Number of Deuterons
Time, minutes
9-19
9-20
9-21
9-22
12-19
12-21
12-22
13-21
14-21
16-21
20-32
20-34
22-33
22-35
22-39
22-44
23-33
23-39
23-44
26-34

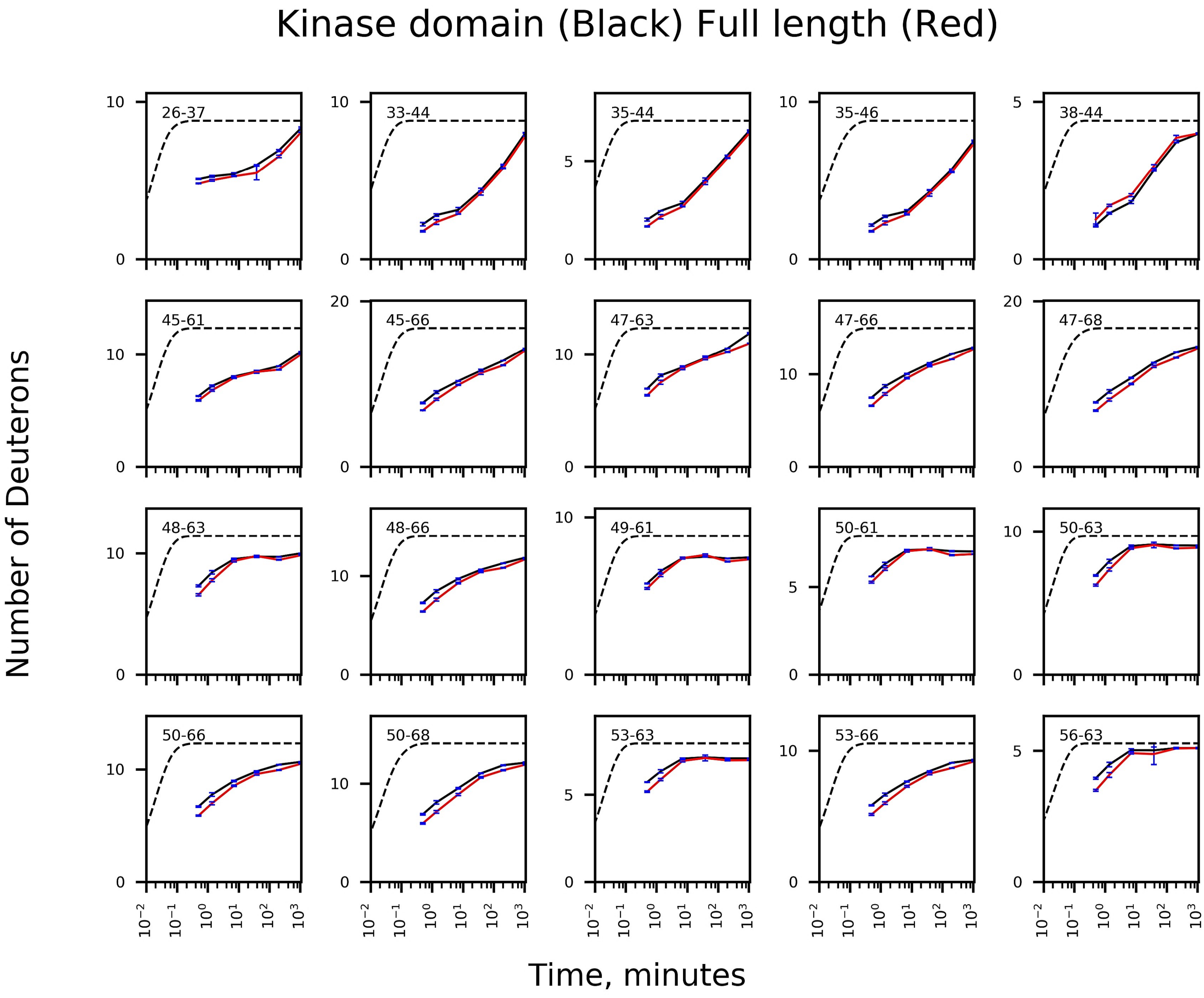

Kinase domain (Black) Full length (Red)
Number of Deuterons
Time, minutes
26-37
33-44
35-44
35-46
38-44
45-61
45-66
47-63
47-66
47-68
48-63
48-66
49-61
50-61
50-63
50-66
50-68
53-63
53-66
56-63

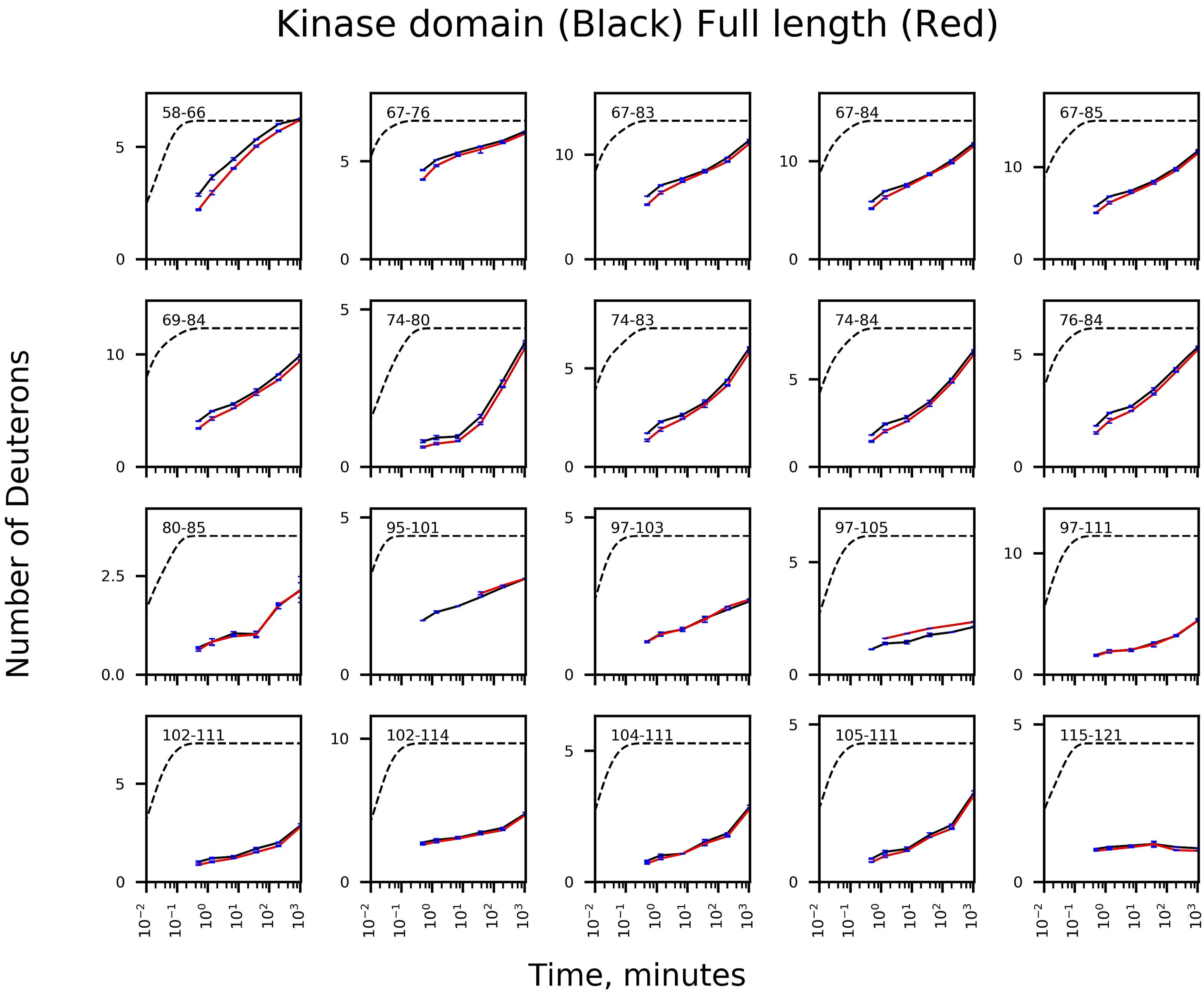

Kinase domain (Black) Full length (Red)
58-66
67-76
67-83
67-84
67-85
69-84
74-80
74-83
74-84
76-84
80-85
95-101
97-103
97-105
97-111
102-111
102-114
104-111
105-111
115-121
Number of Deuterons
Time, minutes

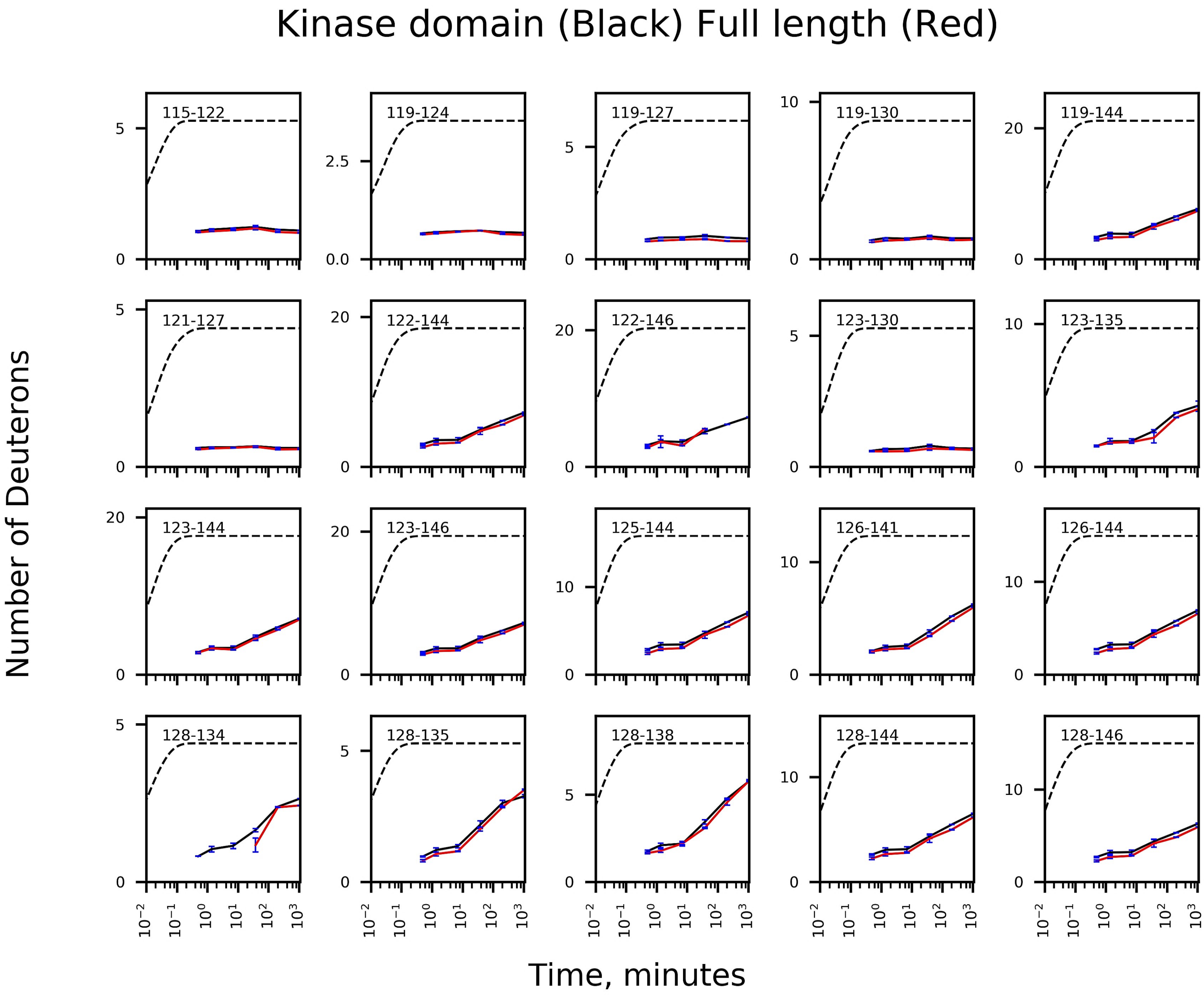

Kinase domain (Black) Full length (Red)
Number of Deuterons
Time, minutes
115-122
119-124
119-127
119-130
119-144
121-127
122-144
122-146
123-130
123-135
123-144
123-146
125-144
126-141
126-144
128-134
128-135
128-138
128-144
128-146

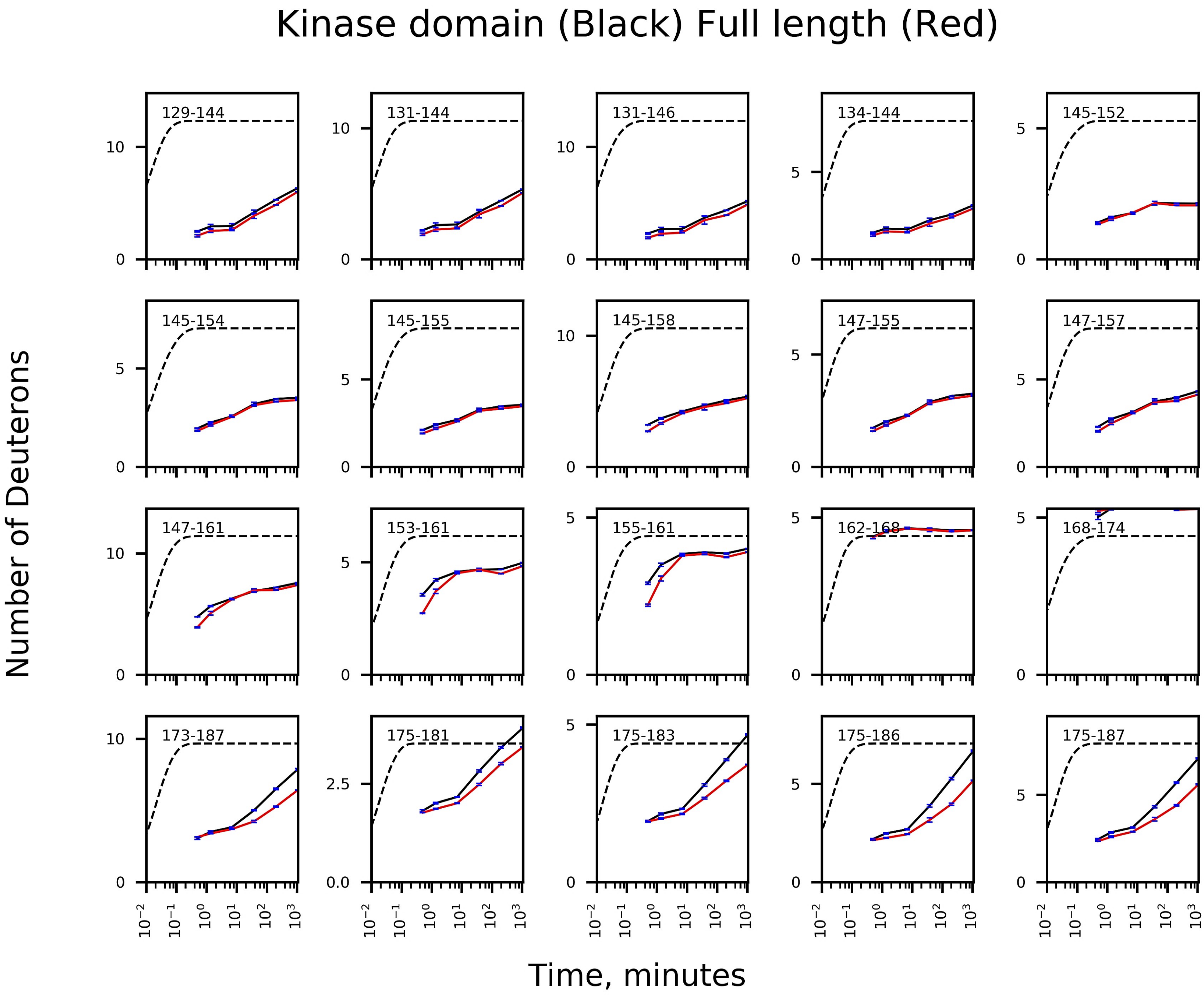

Kinase domain (Black) Full length (Red)
Number of Deuterons
Time, minutes
129-144
131-144
131-146
134-144
145-152
145-154
145-155
145-158
147-155
147-157
147-161
153-161
155-161
162-168
168-174
173-187
175-181
175-183
175-186
175-187

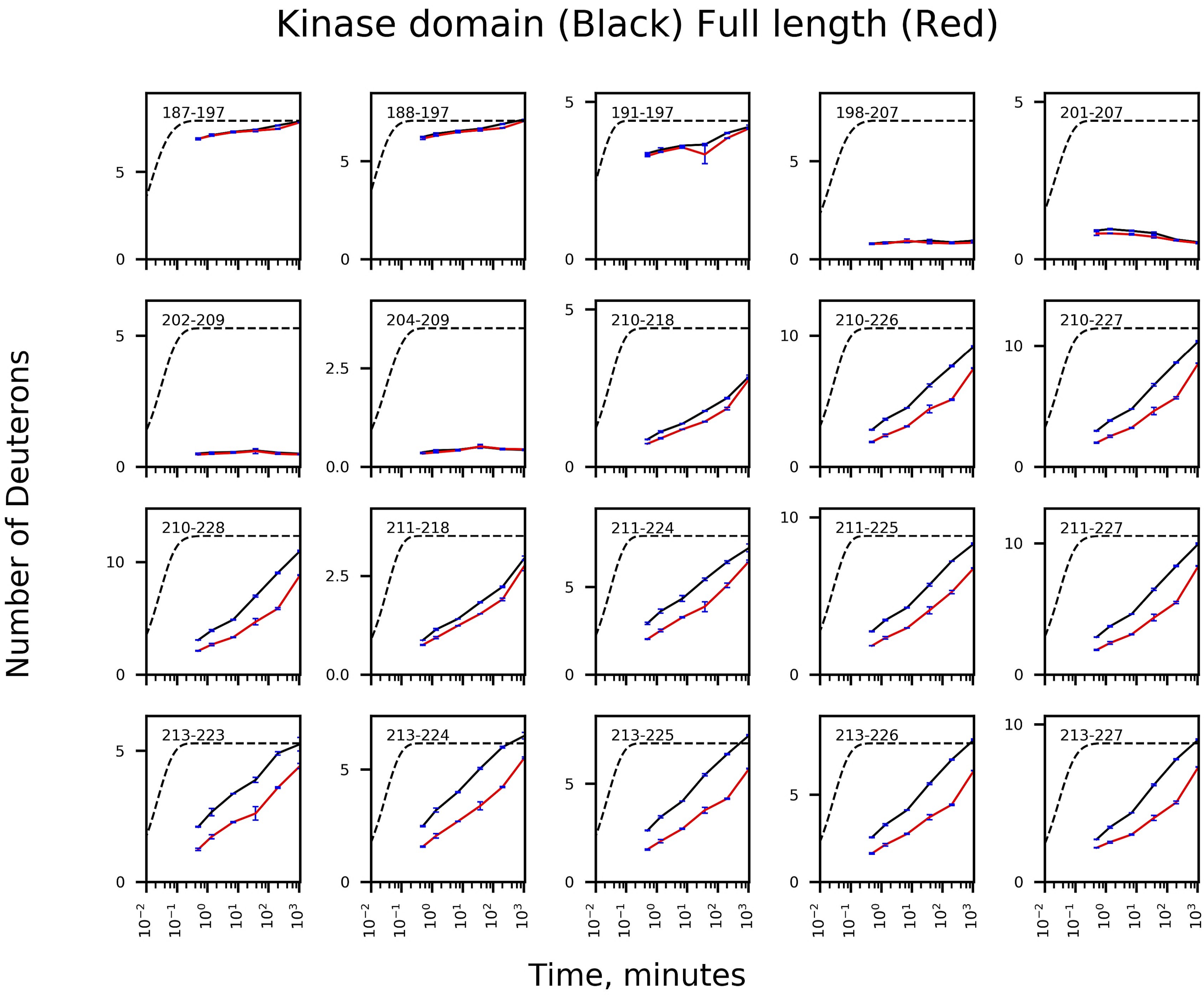

Kinase domain (Black) Full length (Red)
Number of Deuterons
Time, minutes
187-197
188-197
191-197
198-207
201-207
202-209
204-209
210-218
210-226
210-227
210-228
211-218
211-224
211-225
211-227
213-223
213-224
213-225
213-226
213-227

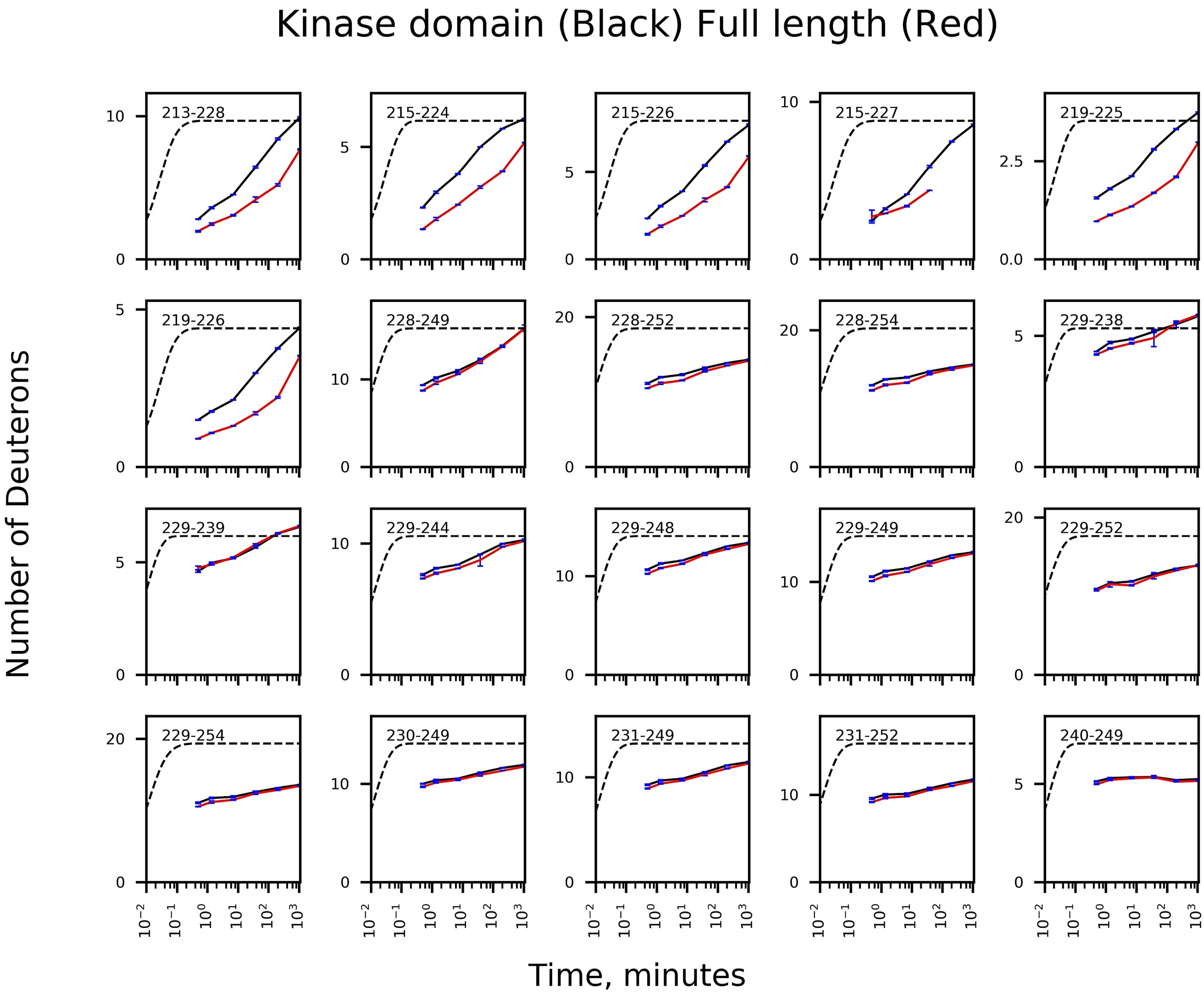
Kinase domain (Black) Full length (Red)
Number of Deuterons
Time, minutes
213-228
215-224
215-226
215-227
219-225
219-226
228-249
228-252
228-254
229-238
229-239
229-244
229-248
229-249
229-252
229-254
230-249
231-249
231-252
240-249

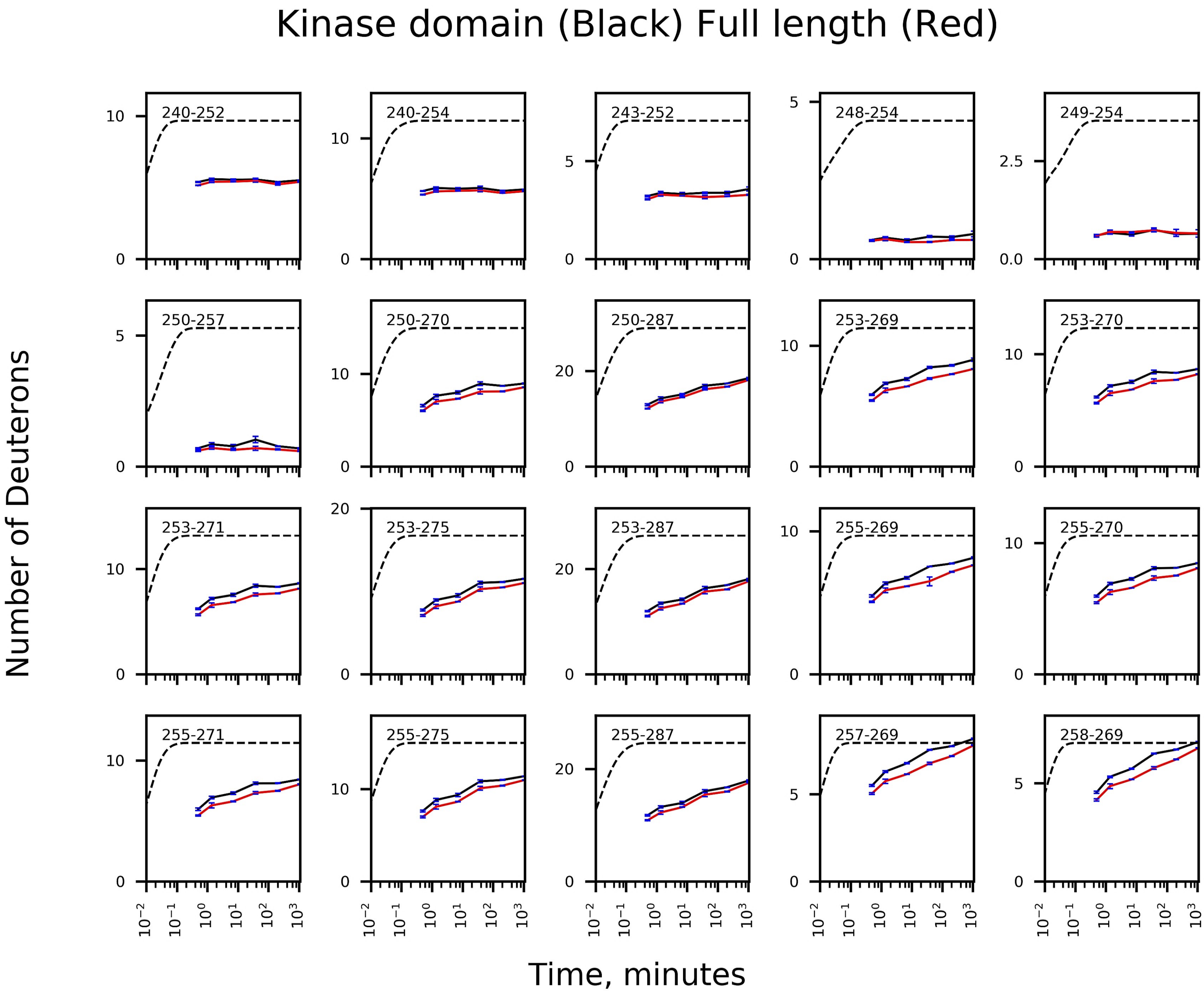

Kinase domain (Black) Full length (Red)
Number of Deuterons
Time, minutes
240-252
240-254
243-252
248-254
249-254
250-257
250-270
250-287
253-269
253-270
253-271
253-275
253-287
255-269
255-270
255-271
255-275
255-287
257-269
258-269

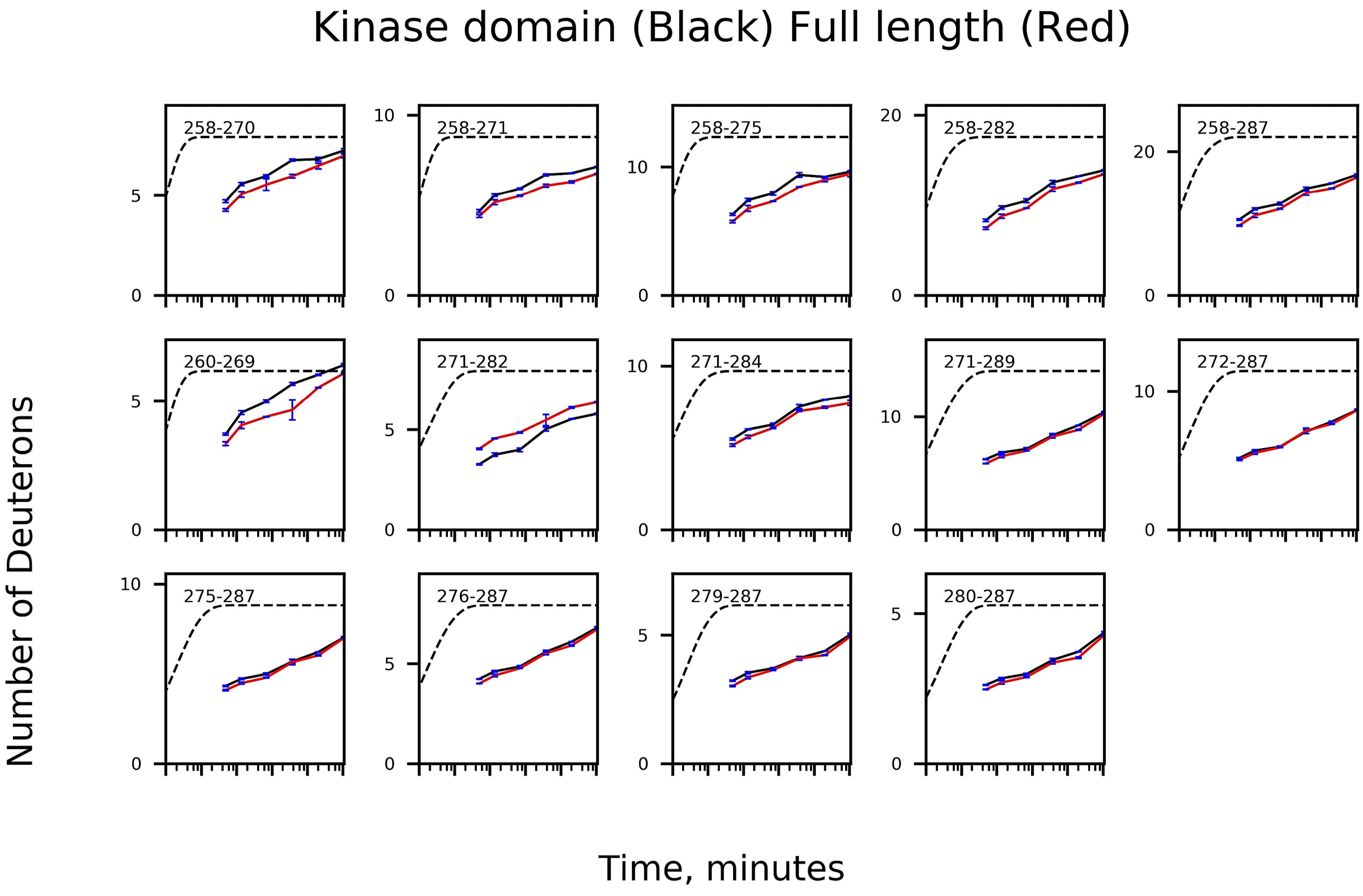
Kinase domain (Black) Full length (Red)
258-270
258-271
258-275
258-282
258-287
260-269
271-282
271-284
271-289
272-287
275-287
276-287
279-287
280-287
Number of Deuterons
Time, minutes
