# Supplementary material for: HPK1 citron homology domain regulates phosphorylation of SLP76 and modulates kinase domain interaction dynamics
Source: Nat Commun. 2024 May 2;15:3725. doi: 10.1038/s41467-024-48014-9 (PMC11066036; doi:10.1038/s41467-024-48014-9)
Supplement: Supplementary file 5 — Supplementary Data 2 [file 41467_2024_48014_MOESM5_ESM.pdf]

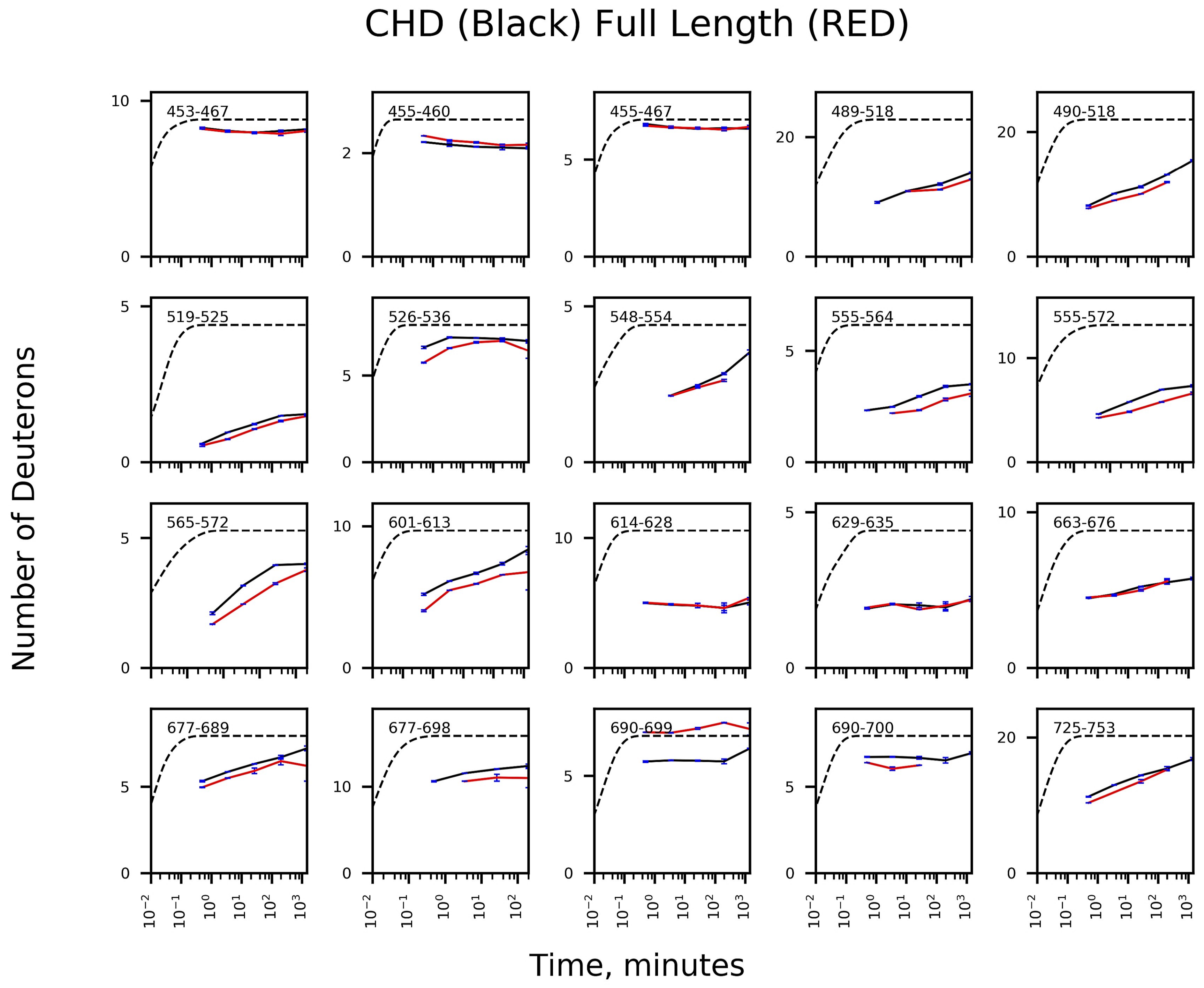
CHD (Black) Full Length (RED)
Number of Deuterons
Time, minutes
453-467
455-460
455-467
489-518
490-518
519-525
526-536
548-554
555-564
555-572
565-572
601-613
614-628
629-635
663-676
677-689
677-698
690-699
690-700
725-753

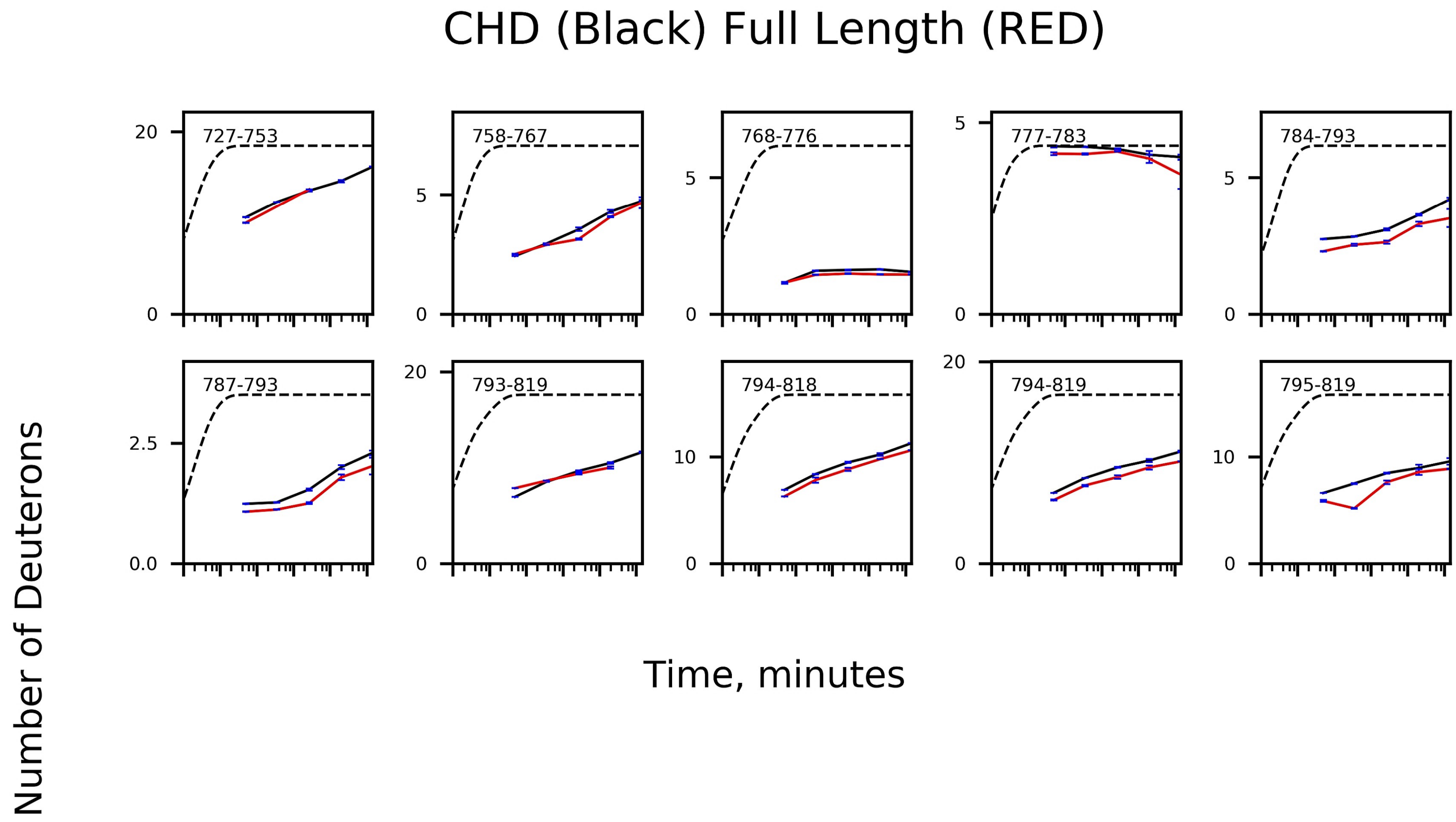

CHD (Black) Full Length (RED)
727-753
758-767
768-776
777-783
784-793
787-793
793-819
794-818
794-819
795-819
Number of Deuterons
Time, minutes
