# Supplementary material for: HPK1 citron homology domain regulates phosphorylation of SLP76 and modulates kinase domain interaction dynamics
Source: Nat Commun. 2024 May 2;15:3725. doi: 10.1038/s41467-024-48014-9 (PMC11066036; doi:10.1038/s41467-024-48014-9)
Supplement: Supplementary file 7 — Reporting Summary [file 41467_2024_48014_MOESM7_ESM.pdf]

nature portfolio

Corresponding author(s): Ira Mellman, Laetitia Comps-Agrar, Weiru Wang

Last updated by author(s): Mar 4, 2024

Reporting Summary

Nature Portfolio wishes to improve the reproducibility of the work that we publish. This form provides structure for consistency and transparency in reporting. For further information on Nature Portfolio policies, see our Editorial Policies and the Editorial Policy Checklist.

Statistics

For all statistical analyses, confirm that the following items are present in the figure legend, table legend, main text, or Methods section.

n/a	Confirmed	
☐	☒	The exact sample size (*n*) for each experimental group/condition, given as a discrete number and unit of measurement
☐	☒	A statement on whether measurements were taken from distinct samples or whether the same sample was measured repeatedly
☐	☒	The statistical test(s) used AND whether they are one- or two-sided<br>*Only common tests should be described solely by name; describe more complex techniques in the Methods section.*
☒	☐	A description of all covariates tested
☐	☒	A description of any assumptions or corrections, such as tests of normality and adjustment for multiple comparisons
☐	☒	A full description of the statistical parameters including central tendency (e.g. means) or other basic estimates (e.g. regression coefficient) AND variation (e.g. standard deviation) or associated estimates of uncertainty (e.g. confidence intervals)
☐	☒	For null hypothesis testing, the test statistic (e.g. *F*, *t*, *r*) with confidence intervals, effect sizes, degrees of freedom and *P* value noted<br>*Give P values as exact values whenever suitable.*
☒	☐	For Bayesian analysis, information on the choice of priors and Markov chain Monte Carlo settings
☒	☐	For hierarchical and complex designs, identification of the appropriate level for tests and full reporting of outcomes
☒	☐	Estimates of effect sizes (e.g. Cohen's *d*, Pearson's *r*), indicating how they were calculated

Our web collection on statistics for biologists contains articles on many of the points above.

Software and code

Policy information about availability of computer code

Data collection	Biacore T200 control software (version 3.2.1 Cytiva), FACS Diva, GloMax Discover System sofware (version 4.0.0), MSconvert GUI (now version 3.0.19256-a8cbe7417), ExMS program version 2, Byonic® (version 3.2, Protein-Metrics Inc.), Byologic® (version 3.2, Protein-Metrics Inc.), cSeries Capture Software v1.9.8.0403, BD FACSDiva v9.1, EnVision® Multilabel Plate Reader, XDS (Version October 15, 2015)
Data analysis	GraphPad Prism 9, Biacore T200 evaluation software (version 3.2.1 Cytiva), LI-COR Image Studio Lite v5.2.5, FlowJo v10.2, Microsoft Excel, Pymol, 2.2.3, Coot, 0.8.7, Phenix & embedded AutoSol 1.9_1692

For manuscripts utilizing custom algorithms or software that are central to the research but not yet described in published literature, software must be made available to editors and reviewers. We strongly encourage code deposition in a community repository (e.g. GitHub). See the Nature Portfolio guidelines for submitting code & software for further information.

Data

Policy information about availability of data

All manuscripts must include a data availability statement. This statement should provide the following information, where applicable:

- Accession codes, unique identifiers, or web links for publicly available datasets
- A description of any restrictions on data availability
- For clinical datasets or third party data, please ensure that the statement adheres to our policy

The crystallographic data for HPK1 CHD have been deposited into the Protein Data Bank under accession codes 8EEC[https://www.rcsb.org/structure/8EEC]. The mass spectrometry data generated in this study, along with sequence files and processed results have been deposited in the MassIVE repository under accession

code MSV000094133 [doi:10.25345/C5707X03Z]. All other data supporting the findings of this study are available within the article and its supplementary files. Any additional requests for information can be directed to the corresponding authors. Source data are provided as a Source Data file with this paper. Source data are provided with this paper.

Research involving human participants, their data, or biological material

Policy information about studies with human participants or human data. See also policy information about sex, gender (identity/presentation), and sexual orientation and race, ethnicity and racism.

Reporting on sex and gender	N/A
Reporting on race, ethnicity, or other socially relevant groupings	N/A
Population characteristics	N/A
Recruitment	N/A
Ethics oversight	N/A

Note that full information on the approval of the study protocol must also be provided in the manuscript.

Field-specific reporting

Please select the one below that is the best fit for your research. If you are not sure, read the appropriate sections before making your selection.

[x] Life sciences [ ] Behavioural & social sciences [ ] Ecological, evolutionary & environmental sciences

For a reference copy of the document with all sections, see nature.com/documents/nr-reporting-summary-flat.pdf

Life sciences study design

All studies must disclose on these points even when the disclosure is negative.

Sample size	Sample sizes were not predetermined for this study. For all cell based assays, 3 independent experiments, at a minimum in duplicates were performed, as further detailed in the Fig legends. The sample size was chosen based on the level of variability across samples and taking into consideration the time-sensitive experimental set up. For SPR assays, at least 4 individual sets of data were generated, as further detailed in the figure legends. The sample size for SPR was chosen to ensure the observed binding response differences between the WT KD (or WT CHD) and their mutants are above assay variation.
Data exclusions	Data were rarely excluded. If they were, it was because one of the replicates looked very different from the others. In any case, the data represent the average of at least 2 duplicates.
Replication	All cell-based assays and SPR experiments (with the exception of the experiments in Fig. 2C, 2E, 3D, S3B-D, S6B-C, S7A, S9 and S10) were performed at a minimum in 3 independent biological replicates as detailed in the respective figure legends. Replication was successful in all cases.
Randomization	*Describe how samples/organisms/participants were allocated into experimental groups. If allocation was not random, describe how covariates were controlled OR if this is not relevant to your study, explain why.*
Blinding	*Describe whether the investigators were blinded to group allocation during data collection and/or analysis. If blinding was not possible, describe why OR explain why blinding was not relevant to your study.*

Reporting for specific materials, systems and methods

We require information from authors about some types of materials, experimental systems and methods used in many studies. Here, indicate whether each material, system or method listed is relevant to your study. If you are not sure if a list item applies to your research, read the appropriate section before selecting a response.

Materials & experimental systems

n/a	Involved in the study
☐	☒ Antibodies
☐	☒ Eukaryotic cell lines
☒	☐ Palaeontology and archaeology
☒	☐ Animals and other organisms
☒	☐ Clinical data
☒	☐ Dual use research of concern
☒	☐ Plants

Methods

n/a	Involved in the study
☒	☐ ChIP-seq
☐	☒ Flow cytometry
☒	☐ MRI-based neuroimaging

Antibodies

Antibodies used

Anti-Human CD3, eBioscience, #14-0037-82, Clone OKT3, Lot 2067619, Final Concentration 10 ug/ml
Anti-Human CD3, Invitrogen, #16-0037-38, Clone OKT3, Lot 2280254, Final Concentration 10 ug/ml
AffiniPure goat anti-mouse IgG, Jackson ImmunoResearch. #115-005-062, Lots 146203/146955, Final Concentration 10 ug/ml
Anti-Human CD28, Invitrogen, #16-0289-85, Lot 2121948, Clone CD28.2, Final Concentration 5 ug/ml
Anti-rabbit IgG (H+L), F(ab')2 Fragment (PE Conjugate), Cell Signaling, #8885, Dilution 1:1000
HPKl Antibody, Cell Signaling, #4472, Dilution 1:200
HPKl Antibody, Santa Cruz Biotechnology, sc-376169, Clone C-9, Dilution 1:100
Anti-HA-Biotin, Roche, Millipore Sigma, #12158167001, Clone 3F10, Dilution 1:500
B-Actin-HRP, Cell Signaling, #5125, Clone 13E5, Dilution 1:5000
Anti-KO Antibody Fab used in SPR assay was produced at Genentech
Anti-Phosphorylated SLP76 (5376) used in Flow Cytometry assays was produced at Genentech, Final Concentration 2 ug/ml
HTRF Mab anti-Flag M2-d2, Revvity, #61FG2DL
All above references are added to the main text.

Validation

Validation of commercial antibodies used in this study were done by the manufacturer and details of these validation efforts are described on the manufacturer's website.

Eukaryotic cell lines

Policy information about cell lines and Sex and Gender in Research

Cell line source(s)

Jurkat.HPK1 KO.Clone 6-5 (Jurkat cell line engineered in house using a CRISPR approach to knock out HPK1, cloned by limiting dilution). References added to the main text.

Authentication

Cell line was not authenticated

Mycoplasma contamination

Cell line was tested and was negative for mycoplasma contamination

Commonly misidentified lines
(See ICLAC register)

No commonly misidentified lines were used.

Plants

Seed stocks

Report on the source of all seed stocks or other plant material used. If applicable, state the seed stock centre and catalogue number. If plant specimens were collected from the field, describe the collection location, date and sampling procedures.

Novel plant genotypes

Describe the methods by which all novel plant genotypes were produced. This includes those generated by transgenic approaches, gene editing, chemical/radiation-based mutagenesis and hybridization. For transgenic lines, describe the transformation method, the number of independent lines analyzed and the generation upon which experiments were performed. For gene-edited lines, describe the editor used, the endogenous sequence targeted for editing, the targeting guide RNA sequence (if applicable) and how the editor was applied.

Authentication

Describe any authentication procedures for each seed stock used or novel genotype generated. Describe any experiments used to assess the effect of a mutation and, where applicable, how potential secondary effects (e.g. second site T-DNA insertions, mosiacism, off-target gene editing) were examined.

Flow Cytometry

Plots

Confirm that:

- [x] The axis labels state the marker and fluorochrome used (e.g. CD4-FITC).
- [x] The axis scales are clearly visible. Include numbers along axes only for bottom left plot of group (a 'group' is an analysis of identical markers).
- [x] All plots are contour plots with outliers or pseudocolor plots.
- [x] A numerical value for number of cells or percentage (with statistics) is provided.

Methodology

Sample preparation	HPK1-/- Jurkat cultured cell line (Clone 6.5, developed in-house) was used.
Instrument	BD FACSymphony A3 Dual
Software	BD FACS Diva v9.1
Cell population abundance	Cells were sorted to purify populations containing the GFP-tagged constructs. Purity of sorted samples was checked by assessed by analyzing the sample post-sort, where purity was >99%.
Gating strategy	For sorting of cells expressing retroviral constructs, cells were stained with eBioscience Fixable Viability Dye eFluor 780 and flow cytometry was performed . The gating strategy is outlined in Suppl. Fig. X. Briefly, cells were gated based on FCS-A/SSC-A and singlets identified by FSC-H/FSC-A gating, followed by gating on live (eFluor 780-) cells. Retrovirus-containing cells were gated on GFP positive cells and sorted.<br><br>For determination of phosphorylated SLP76 (pSLP76) levels by flow cytometry, cells were stimulated, fixed, and stained as described in the methods and run on a flow cytometer. The gating strategy is outlined in Suppl. Fig. S7. Briefly, cells were gated based on FCS-A/SSC-A and singlets identified by FSC-H/FSC-A gating, followed by gating on live (eFluor 780-) cells. Amount of pSLP76 was determined by quantification of PE positive (detection of PE-conjucated secondary antibody to in-house Rabbit anti-pSLP76 antibody) cells.

- [x] Tick this box to confirm that a figure exemplifying the gating strategy is provided in the Supplementary Information.
